# Exploring inhomogeneous surfaces: Ti-rich SrTiO_3_(110) reconstructions *via* active learning†

**DOI:** 10.1039/d4dd00231h

**Published:** 2024-09-16

**Authors:** Ralf Wanzenböck, Esther Heid, Michele Riva, Giada Franceschi, Alexander M. Imre, Jesús Carrete, Ulrike Diebold, Georg K. H. Madsen

**Affiliations:** a Institute of Materials Chemistry, TU Wien 1060 Vienna Austria georg.madsen@tuwien.ac.at; b Institute of Applied Physics, TU Wien 1040 Vienna Austria; c Instituto de Nanociencia y Materiales de Aragón, CSIC-Universidad de Zaragoza 50009 Zaragoza Spain

## Abstract

The investigation of inhomogeneous surfaces, where various local structures coexist, is crucial for understanding interfaces of technological interest, yet it presents significant challenges. Here, we study the atomic configurations of the (2 × *m*) Ti-rich surfaces at (110)-oriented SrTiO_3_ by bringing together scanning tunneling microscopy and transferable neural-network force fields combined with evolutionary exploration. We leverage an active learning methodology to iteratively extend the training data as needed for different configurations. Training on only small well-known reconstructions, we are able to extrapolate to the complicated and diverse overlayers encountered in different regions of the inhomogeneous SrTiO_3_(110)-(2 × *m*) surface. Our machine-learning-backed approach generates several new candidate structures, in good agreement with experiment and verified using density functional theory. The approach could be extended to other complex metal oxides featuring large coexisting surface reconstructions.

## Abstract 2

The atomic configurations of the inhomogeneous surfaces are unraveled using an evolutionary strategy backed by a machine-learned neural-network force field. Excellent agreement with scanning tunneling microscopy images is demonstrated.

## Graphical abstract

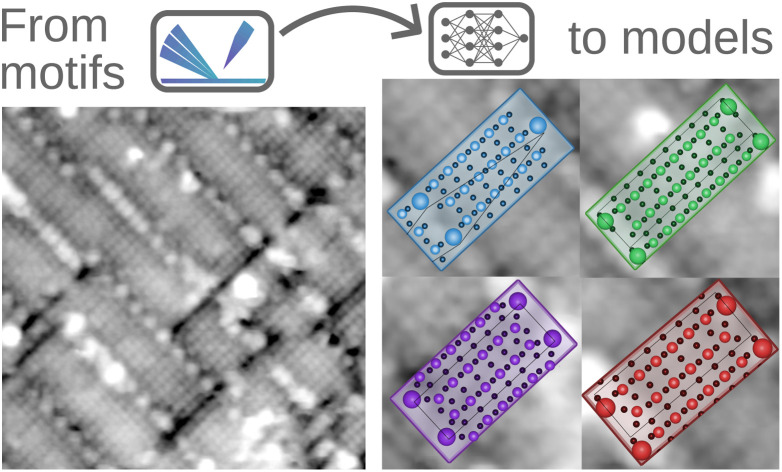

## 1 Introduction

The surfaces of metal oxides play key roles in countless natural processes and technological applications. At the atomic level, the properties of metal oxide surfaces, such as reactivity, electronic structure, and defect formation, are intricately linked to their performance. Only through a deep understanding of these surfaces at the atomic scale, can their properties be precisely controlled and optimized.

Machine-learned force fields (MLFFs) are an increasingly popular tool that can be used to explore surface structures, including those of metal oxides. Method development and their applications have been advancing in parallel, with innovative and powerful models synergizing with established and proven methods. For example, moving from neural-network force fields that utilize precomputed invariant descriptors^1–3^ to adopting equivariant message passing networks^4–6^ has enabled more data-efficient and transferable MLFFs. Modern applications include foundation models trained on a wide range of materials,^7^ transferable water potentials,^8^ and condensed phase chemistry.^9^

Here, we utilize MLFFs to explore the surface reconstructions of strontium titanate (SrTiO_3_), a perovskite oxide used as a model system for the development of many technological applications, including optoelectronics, catalysis, memory devices, and photovoltaics.^10–13^ SrTiO_3_ is also exciting from a fundamental standpoint: It exemplifies the richness of bulk, surface, and interface properties that can be accessed within a single perovskite material: donor doping by chemical impurities,^14,15^ oxygen vacancies,^14,16,17^ or field effects^15,18^ can turn it into an insulator, a metal, a superconductor or even induce confined metallic behaviour in the form of 2D electron gases. The diversity extends to the atomistic details of the surface,^19^ where a variety of composition-related, polarity-compensating reconstructions have been found for the (001), (110), and (111) orientations.^20–27^ Pinpointing the regions of stability of such reconstructions and gaining a deep understanding of the atomic-scale details of their surfaces is essential for designing SrTiO_3_-based systems with tailored functionalities.

Many studies exist that explore the atomic details of the surface of single-crystalline SrTiO_3_ samples under ultra-high vacuum (UHV) conditions. It is known that specific surface reconstructions are difficult to reproduce and can depend on sample history and preparation conditions.^19^ Notably, scanning tunneling microscopy (STM) studies of SrTiO_3_ surfaces frequently reveal the coexistence and even intermixing of multiple surface structures,^21,26,28^ a feature common to the surfaces of other complex perovskites such as BaTiO_3_ and (La, Sr)MnO_3_ (ref. 29 and 30). The variety of coexisting surface reconstructions and their dependence on sample history underlines the necessity of changing the framing from identifying a single specific reconstruction to mapping out the range of possible reconstructions. This diversity can serve as an ideal showcase of the power of MLFF-supported stochastic searches, which, in turn, could be applied to the exploration of surface structures of further, technologically relevant materials.

In particular, the enhanced accuracy and reliability of MLFFs facilitate the application of stochastic algorithms for structural exploration of materials.^31–36^ Stochastic approaches require a substantial volume of calculations and are impracticable with *ab initio* methods such as density functional theory (DFT) as the backend. This holds especially true for large and complex systems, such as surface reconstructions of multi-element compounds. Such systems usually feature a complex energy surface with too many degrees of freedom to explore exhaustively, as well as many local minima, where local searches for an optimal structure largely depend on the initial geometry of the search. Given that stochastic searches produce more diverse structures than, *e.g.*, molecular dynamics, a transferable, robust, and generalizable force field trained on a diverse dataset is key to their success.

An accurate MLFF is, however, just one part of the toolbox necessary to build a robust and efficient workflow for structure searches. The design of an MLFF can enhance or restrict its transferability and any MLFF has the potential to emit infinitely diverse mispredictions. This is especially significant for stochastic searches, which, by design, tend to move into regions that the model was not trained on. Extrapolation happens almost surely in high-dimensional models,^37^ and therefore is not, by itself, an indicator of poor performance. Research has hence focused on estimates of uncertainty as proxies for the error incurred by using a given MLFF.^38–42^ When that error is suspected to exceed tolerable margins, retraining with an expanded training set can help extend the applicability of the MLFF. In such scenarios, an efficient algorithm must aim toward issuing only as many *ab initio* calculations as required, while preventing waste of resources on redundant or irrelevant configurations.^43,44^ Thus, the need for an uncertainty metric to evaluate the quality of the results dovetails with the usefulness of such metrics for identifying or even generating optimally informative new configurations.^33^ This makes stochastic structure searches naturally part of the domain of application of active learning (AL) workflows, where an informed data selection is achieved through uncertainty estimation.^45^

**Fig. 1:**
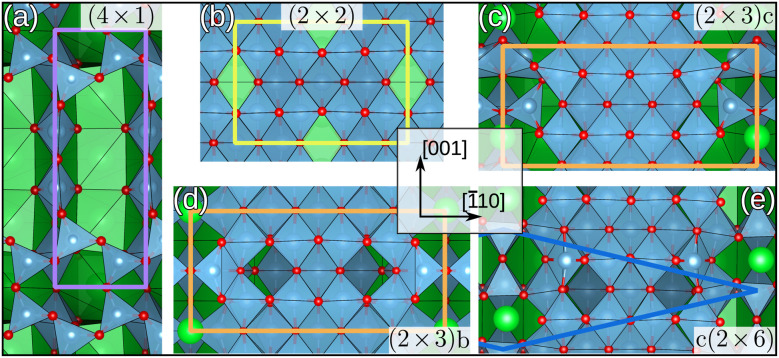
Top view of SrTiO_3_(110)-(*n* × *m*) surface reconstructions that are used for MLFF training: previously explored (4 × 1),^22^ (2 × 2) and (2 × 3)b,^25^ and newly identified (2 × 3)c and c(2 × 6) from this work. O atoms are shown in red, Sr in green and Ti in blue. The blue polyhedra depict TiO_*x*_ polyhedra in all panels. Colored lines indicate the borders of unit cells. For (e) only half the unit cell is outlined. See Fig. S10 of the ESI† for the full unit cell and additional side views. (*n* × 1) overlayers exclusively contain tetrahedrally coordinated TiO_4_ units, while SrTiO_3_(110)-(2 × *m*) surface reconstructions are predominantly composed of octahedrally coordinated TiO_6_ units and in experimental observations include at least one Sr atom per unit cell.^19,25^

This work focuses on the less well-understood Ti-rich surface reconstructions at the (110) orientation of SrTiO_3_. Literature describes numerous composition-related SrTiO_3_(110) surface reconstructions that can be broadly grouped into two families, characterized by Ti-poor (*n* × 1) and Ti-rich (2 × *m*) overlayers on an otherwise unchanged bulk.^26^ Here, *n* and *m* denote the number of (1 × 1) bulk unit cells covered in the [001] and [1̄10] directions, respectively. Fig. 1 shows examples of both variations. Ti-rich overlayers pose the particular challenge that, even when the Ti-to-Sr ratio is controlled,^26^ numerous reconstructions lacking corresponding DFT models can coexist. Here, we again observe pronounced inhomogeneity in new STM measurements and are able to identify varied reconstructions (see Fig. S1 of the ESI†). We mitigate the lack of suitable atomistic models of such inhomogeneous surfaces by combining an evolutionary search algorithm with a transferable MLFF to identify valid candidate structures. Transferability, in particular, is an important prerequisite for minimizing the computational cost associated with generating training data. We demonstrate that by utilizing small, well-known reconstructions and implementing a careful data selection routine built on structural and spatially-resolved local uncertainties, we can arrive at an MLFF capable of extrapolating to larger, more complex structures. In the following, we first discuss the active learning approach. We then proceed to show how structural models reproducing the STM images can be systematically obtained for all the coexisting surface structures.

## 2 Approach

### 2.1 Active learning workflow for the initial dataset

We set out to iteratively construct a versatile MLFF, which can subsequently be used in combination with an evolutionary search, specifically the covariance matrix adaptation evolution strategy (CMA-ES),^46^ to explore the different motifs encountered on the inhomogeneous, Ti-rich SrTiO_3_(110) surface. In this study, we refer to the suggested atomistic surface reconstruction models as “unit cells” and to the corresponding regions of the STM images as “motifs”. Due to the complex nature of the various surface reconstructions, the MLFF must be capable of resolving a wide range of local environments. Additionally, given the nature of the evolutionary search, which produces more diverse and possibly unphysical structures than, *e.g.*, molecular dynamics, intermediate configurations are likely to exhibit unusual properties, such as unphysical bond lengths. For that reason, the MLFF needs to be robust and the underlying training data diverse, making the construction of a suitable training set containing *ab initio* energies and forces far from trivial. Furthermore, we aim to explore (2 × 5) surface reconstructions with up to 450 atoms per unit cell, where the computational costs associated with DFT evaluation is prohibitive for constructing diverse training databases. The MLFF must therefore also be able to generalize from smaller training structures to the larger unit cells explored.

**Fig. 2:**
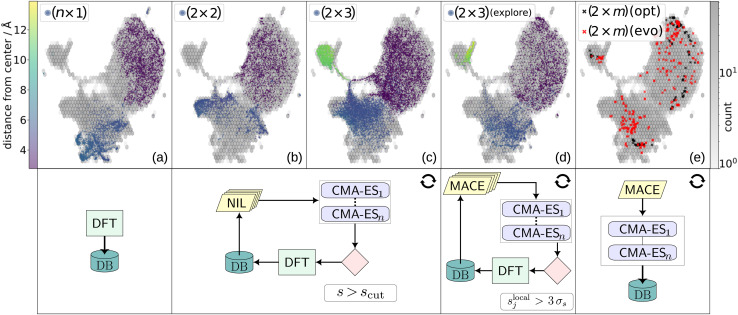
The upper row shows a two-dimensional UMAP representation of the spherical Bessel descriptors of the local environments of Sr atoms. The background of all plots displays the training data as fitted utilizing a log–log hexbin approach, with darker shading indicating higher data density per bin (right color bar). In the foreground of the first four columns, colored dots depict the local environments of Sr atoms corresponding to the structures labeled (*n* × 1), (2 × 2), (2 × 3)  and “(2 × 3) (explore)”, respectively. Here, the colors indicate each atom's distance from the center of the surface slab (left color bar). Column (e) features the local Sr descriptors of geometry-optimized (2 × *m*) results (including c (2 × 6), (2 × 4)c, c(2 × 8), (2 × 4)d, (2 × 4)f, (2 × 5)b, and (2 × 5)c projected on the same 2D UMAP background as black crosses. The red crosses in the same subplot depict the same for randomly chosen individuals from earlier generations of (2 × 4)f evolution runs, including generations 10, 25, and 50. In the bottom row, the active learning workflow is schematically illustrated. The circle arrows represent iterations, the stacked parallelograms depict MLFF committees, and the CMA-ES blocks indicate parallel and/or sequential execution of multiple CMA-ES runs.

We started from the basis of our previous results on a Ti-poor reconstruction of SrTiO_3_(110), namely the (4 × 1) (see Fig. 1(a)).^35^ There, overlayers were symmetrically set up on opposite sides along the surface normal, attached to bulk-like layers in between. All structures in this study are constructed in the same manner as introduced in ref. 35. We constructed a database of 495 structures, re-evaluated using VASP^47^ with the r^2^SCAN functional.^48^ This initial DFT database is illustrated in Fig. 2 together with a 2D projection of the spherical Bessel descriptors of the local environments of Sr atoms obtained using the uniform manifold approximation and projection (UMAP) method for dimension reduction.^49^

Using these data we trained a ten-member committee based on the descriptor-based NeuralIL architecture.^3,39^ In committees the uncertainty is approximated by training a set of models that vary by initialization seed, hyperparameters, architecture, or training data, and monitoring their disagreement on a prediction to obtain the model variance. The majority of the computational cost incurred when training a descriptor-based model can be attributed to the calculation of the descriptors and the associated vector-Jacobian product operator. In this study, we vary the initial seed to enable NeuralIL's particularly efficient committee implementation that reuses descriptor encodings for all members, so that committees needed for uncertainty estimation can be trained with a negligible performance penalty.^39^

We then generated CMA-ES trajectories for the (2 × 2), (2 × 3)a and (2 × 3)b reconstructions, see Fig. 1. The CMA-ES^46^ samples a population of *λ* individuals, *x*^(*g*)^_k_, *k* = 1, …, *λ*, for every generation *g* from the multivariate normal distribution1
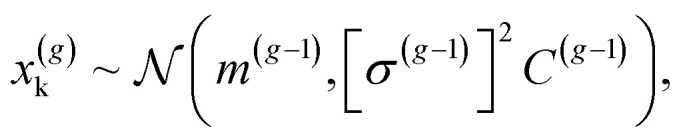
with distribution mean *m*, step size *σ* and covariance *C*. In the present case, the *x*^(*g*)^_k_ are the Cartesian coordinates of the atoms as explained in the Technical details section. The population size *λ*, initial mean *m*^(0)^, and initial step size *σ*^(0)^, are user-defined hyperparameters. We refer to the initial mean as the founder structure and data generation started from founders that are variations of published structures.^25^ The mean is updated to move the average towards individuals/structures with low energy. Similarly, the covariance matrix is updated to let it reflect successful steps according to the CMA-ES algorithm.^46^

We then iteratively added structures from the MLFF backed CMA-ES trajectories using the committee uncertainty estimate aggregated structure-wise^39,42^2
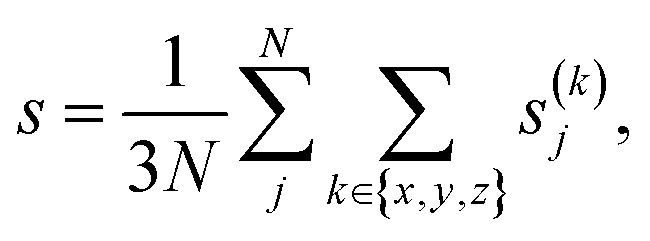
to identify structures that should be added to the training data. Here, *N* is the number of atoms and *s*^(*k*)^_*j*_ the committee standard deviation of the *k*-th component of the force on atom *j*. In total, 519 (2 × 2) and 775 (2 × 3) training structures were generated during this process. The dataset was then refined by incorporating 141 (5 × 1) and a further 296 (4 × 1) structures from ref. 35 into the training data in order to reinforce performance on SrTiO_3_(110)-(*n* × 1) structures.

The structures added to the training data are illustrated in Fig. 2 (top row) using the 2D UMAP projection. The background shows the distribution of all SrTiO_3_(110) data from the final dataset through hexagonal binning. The foreground of subplots (a)–(c) depicts the local Sr environments of the individual (*n* × 1), (2 × 2) and (2 × 3) data sets, with the colors indicating the distance of each Sr atom from the center of the surface slab. It can be seen how the data from each reconstruction used for the initial dataset contributes their own distinct environments. Not surprisingly, the Sr in the (2 × 3) overlayer reconstructions, depicted as green points in the top left of Fig. 2(c), is particularly distinctive, since it did not occur in the (*n* × 1) or (2 × 2) training data. In the bottom row of Fig. 2, the workflows used for generating the data depicted directly above them are illustrated, with (b) and (c) generated by different iterations of the same workflow.

### 2.2 Exploration-based active learning

Using the 2226 structures in the training database, we trained a five-member committee using the equivariant message-passing neural network framework MACE.^4^ MACE provides significantly improved accuracy and transferability and is more data-efficient than NeuralIL due to its equivariant architecture and custom-learned atomic representations. The mean absolute error in the force components, *f*_MAE_, for the (4 × 1), (2 × 2) and (2 × 3) sets decreased by a factor of 2.5 when moving from NeuralIL to MACE with the same training data (see Table S2†). Notably, the aggregated force uncertainty estimates obtained from MACE and NeuralIL committees, eqn (2), exhibit strong correlations for all highly uncertain configurations (see Fig. S3 of the ESI†). This underscores the efficiency of constructing the database by using uncertainties derived from the NeuralIL committee. The initial MACE model demonstrated strong performance on test data and could reliably be applied to investigate (*n* × 1) and (2 × 2) structures using the CMA-ES with relatively large population sizes, *λ* = 100, and initial step sizes, *σ*^(0)^ ∈ [0.1, 0.35].

We then performed exploratory CMA-ES searches on the (2 × 3) surface with population size *λ* = 100, and varying the initial step size in the range of *σ*^(0)^ ∈ [0.1, 0.5]. To expand the search space, we developed a more generic founder structure (pictured in Fig. S7 of the ESI†), rather than relying on published findings. From these searches, we identified the new SrTiO_3_(110)-(2 × 3)c reconstruction, Fig. 1(c). A key feature of this structure is the alignment of the overlayer Sr atoms relative to the topmost TiO_*x*_ rows. Geometry optimizing the (2 × 3)b and (2 × 3)c structures using VASP, reveals that the new (2 × 3)c has a lower energy of Δ*E* = 160 meV per (1 × 1) bulk unit cell.

**Fig. 3:**
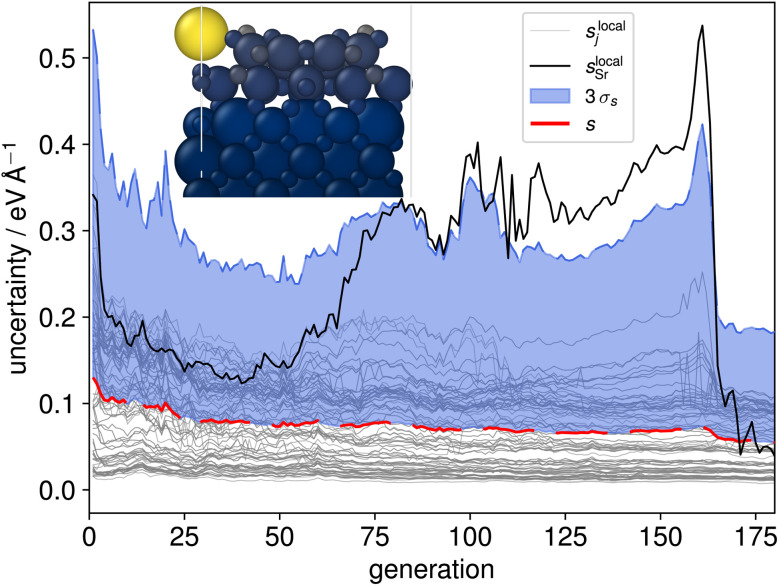
Spatially resolved uncertainty of a CMA-ES trajectory of a mirror-symmetric SrTiO_3_(110)-(2 × 3) structure. The gray lines show the locally aggregated uncertainty *s*^local^_j_ of each atom. The mean of all local uncertainties, *i.e.*, the global structure uncertainty *s*, is depicted as a dashed red line, with three times their standard deviation *σ*_s_ shaded in blue. The local uncertainty associated with the overlayer Sr atom is highlighted in black. The inset shows a structure at generation 160 with the atoms colored according to the local uncertainty estimate. The color scale ranges from dark-blue (lowest) to yellow (highest local uncertainty).

While two of the 35 initial CMA-ES searches produced the new stable configuration, a majority of these evolutionary searches were found to be prone to instability, specifically to the expulsion of an Sr atom (see inset structure in Fig. 3). Although problematic structures could be identified manually, an active learning procedure needs to be able to identify and incorporate such structures into the training data based on computed quantities such as model uncertainties. Interestingly this behaviour was not reflected in the aggregated structure uncertainty *s*, eqn (2), as shown with the red line in Fig. 3. We therefore calculated spatially resolved atomic uncertainties by aggregating over neighboring atoms within a cutoff radius^42^ (in the following set to 4 Å) instead of over the entire structure3
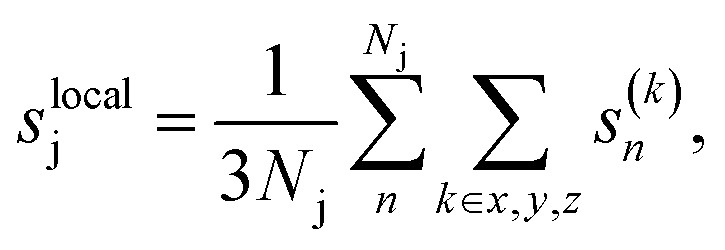
for atom j with *N*_j_ neighbors. Recently, such locally aggregated uncertainties and errors were shown to have a direct monotonic correlation.^42^ This is in contrast to the uncertainties and errors of the individual force components, *s*^(*k*)^_j_, which only feature an asymmetric relationship, where large errors occur predominantly for large uncertainties. However, large uncertainties do not necessitate large errors, causing many false positives when trying to select high-energy structures.^42^ Locally aggregated uncertainties thus allow us to reliably identify high-error sub-regions without false positives.

The local uncertainties, eqn (3), clearly identified the misinterpretation of unphysical local structures which led to escalating errors during the evolution and are thus a reliable indicator for atoms being expelled from the surface. A visual representation of the evolution of local and global uncertainty within a CMA-ES run is given in Fig. 3. The solid black line tracks the local uncertainty associated with the single Sr atom in the overlayer, *s*^local^_Sr_. Notably, *s*^local^_Sr_ begins to increase after generation 70 and exceeds three times the standard deviation of the local uncertainties (3*σ*_s_) after generation 90. This behavior is further illustrated by the atomic structure shown in the inset: at generation 160 even the configuration with the lowest energy features a bright yellow sphere, indicating the high local uncertainty in the force estimate for the Sr atom. The rise in local uncertainty corresponds to environments that the model is increasingly uncertain about but which are localized enough for them to have a low weight in the globally aggregated uncertainty, eqn (2). Once an atom is separated from the rest by more than the cutoff radius of the underlying MLFF model, its contribution to the energy and forces, and thus also to the local and global uncertainty, becomes zero.

In an additional AL step we then identified trajectories where the local uncertainty associated with at least one atom j exceeded three standard deviations of all local uncertainties (*s*_j_^local^ > 3*σ*_s_). We randomly sampled structures from 40 CMA-ES evolution trajectories, with 32 of these structures exhibiting similar local uncertainty behaviour as shown in Fig. 3. The sampling was performed uniformly but was restricted to intact surface slabs, meaning that generations following Sr expulsion were excluded. The 2D UMAP of the local Sr descriptors within these structures is shown in Fig. 2(d), highlighting the added diversity that was achieved. With these additional 392 structures, the full training set consisted of 2618 configurations. This was used to train the final MACE model which was utilized for all further CMA-ES runs. The complete database and trained model are made available on Zenodo.^50^

## 3 Technical details

### 3.1 CMA-ES

We applied CLINAMEN2,^51^ a functional-style Python framework that interfaces to different codes for loss evaluation in a straightforward manner, to perform the covariance matrix adaptation evolution strategy (CMA-ES)^46^ for all structure searches in this study.

Surface slabs were set up as illustrated in Fig. S8.† For all system sizes an anchor region of fixed atom positions was defined at the center of each slab, consisting of bulk-like layers that remained unchanged. The Cartesian coordinates of the atoms in the over- and attachment-layers are the degrees of freedom, *i.e.*, the individuals *x*^(*g*)^_k_, sampled from the multivariate normal distribution. Opposite sides of all slabs were made symmetric. Further symmetry elements (*e.g.*, mirror planes) were leveraged where feasible to drastically reduce the number of degrees of freedom in larger unit cells.

### 3.2 DFT

We used VASP^47^ version 6.2.0 with the r^2^SCAN functional^48^ for all *ab initio* calculations in this study, including single-shot structure evaluations for training and test data and geometry optimization of low-energy candidate structures. The energy cutoff was set to 440 eV, the width of Gaussian smearing to 0.02 eV, and EDIFF to 10^−5^. To ensure compatibility of DFT energies and forces calculated for different system sizes, we utilized the optimized *k*-point grid generator by Wang *et al.*, with the minimum distance set to 25 in the PRECALC input.^52^

### 3.3 Machine-learned force fields

All NeuralIL models in this work used *r*_cut_ = 4.0 Å and *n*_max_ = 5, with ResNet core widths set to [256, 128, 64, 32, 32, 32, 16]. Training on forces was run for only 101 epochs due to the replacement of the standard Adam optimizer with the versatile learned optimizer VeLO,^53^ which drastically reduced the number of epochs needed for convergence by orders of magnitude and eliminated the need to set up a learning rate schedule.^39^

With the majority of hyperparameters set to default values, MACE trainings were performed with a cutoff radius *r*_max_ = 4.0 Å and two hidden layers set to 128 channels for scalar and vector properties each. The maximum number of epochs was set to 1200 with an early stopping patience of 50, and energy and force weights of 1 and 100, respectively. Afterwards, training was run for an additional 300 epochs with an increased energy weight of 1000 and an unchanged force weight.

Notably, as mentioned in the workflow description, training and test data are generated in a manner that allows the resulting MLFF to serve as the backend for CMA-ES runs, instead of DFT codes. This means that the MLFF needs to be able to reliably evaluate configurations far from the equilibrium, which is achieved by utilizing the CMA-ES itself for data generation. At each step, training and test data belong to the same distribution.

To eliminate unusable or obstructive data, all structures with force components larger than 500 eV Å^−1^, as well as those that do not converge with the chosen parameters, are excluded.

### 3.4 Evolution details

In total, approximately 3000 exploratory CMA-ES runs were performed on various system sizes and founder structures. For each founder, runs were started for different random seeds to leverage the stochasticity of the method. Population sizes were varied between *λ* ∈ {25, 35, 50, 100}, with the choice of step size *σ* depending on symmetry. Whenever mirror symmetry was enforced, *σ*^(0)^ was capped at 0.35 Å, while evolutionary searches without symmetry were performed for step sizes up to 0.5 Å. The lower limit for *σ*^(0)^ was 0.1 Å for all cases.

The computation of one CMA-ES trajectory starting from a founder containing 450 atoms and running for up to 1000 generations, with population size *λ* = 100, required only between one and three hours on one NVIDIA A40 GPU with 46 GiB memory when utilizing MACE_full_, depending on early stopping. For that reason, it was possible to freely explore various founders to then select highlights for further investigation using DFT.

### 3.5 Experimental methods

SrTiO_3_(110) single crystals (CrysTec GmbH, 0.5 wt% Nb_2_O_5_, 5 × 5 × 0.5 mm^3^, one-side polished, miscut less than 0.3°) were prepared *ex situ* by sonication in heated neutral detergent (3% Extran^®^ MA02, diluted in ultrapure water, 2 × 20 min) and ultrapure water (milli-Q™, 10 min). Subsequent boiling for 10 min in milli-Q™ water removed commonly observed CaO contamination. The samples were then mounted on flag-style, HNO_3_-cleaned Nicrofer^®^ sample plates with Nicrofer^®^ clips, and inserted in a UHV setup comprising three interconnected chambers: (i) a preparation chamber (base pressure below 10^−10^ mbar) equipped with sputtering–annealing facilities and an evaporator for Sr deposition; (ii) an analysis chamber (base pressure below 5 × 10^−11^ mbar) equipped for STM (SPECS Aarhus STM 150), low-energy electron diffraction (LEED) (Omicron SpectaLEED), and X-ray photoelectron spectroscopy (XPS) (nonmonochromatic dual-anode Mg/Al K*α* source, SPECS Phoibos 100 analyzer, normal emission); (iii) a pulsed-laser deposition (PLD) chamber (base pressure below 2 × 10^−9^ mbar).

After a few cycles of sputtering–annealing (6 × 10^−6^ mbar Ar, 1 keV, 5–10 μA, 10 min, followed by 1 h at 1000 °C, 6 × 10^−6^ mbar O_2_), the surface cleanliness was verified through XPS and STM. The surface stoichiometry was then adjusted *via* submonolayer deposition of Sr (*via* molecular-beam epitaxy)^54^ or TiO_2_ (*via* PLD).^55^ The resulting surface periodicity was verified by LEED and STM. The surface presented in this work was obtained starting from a mixed (4 × 1)/(5 × 1) reconstruction. 1.4 ML Ti was deposited in PLD by keeping the sample at 580 °C in a background oxygen pressure of 6 × 10^−6^ mbar O_2_, followed by ramp down at 80 °C min^−1^.

STM images were acquired in constant-current mode with homemade, electrochemically etched W tips. The tips were prepared *in situ* by Ar sputtering (1 μA, 30 min). Voltage (up to 10 V) or current pulses (up to 10 nA) were applied while in tunneling contact to reshape the tip and improve resolution. Positive bias voltages correspond to tunneling into the empty states of the sample.

## 4 Results and discussion

### 4.1 Structure search

The final MACE model described above enabled us to perform a large number of structure searches for SrTiO_3_(110)-(2 × *m*), *m* ∈ {3, 4, 5}, with initial step sizes in the range of *σ*^(0)^ ∈ [0.1, 0.5]. The choice of population size *λ* strongly influences the stability of the evolution trajectories, especially for such rough loss landscapes. Moreover, a larger population size increases the likelihood of identifying the most stable structure, rather than other stable structures nearby on the loss surface. Because of this trade-off, we performed the same searches with population sizes between 25 and 100, as outlined in the Technical details.

The UMAP in the upper row of Fig. 2(e) illustrates the variety of local Sr environments encountered in randomly selected structures chosen from early generations (10, 25, and 50) along these trajectories (red crosses). In comparison, the local environments in geometry-optimized structures of different unit cell sizes are clearly more uniform (black crosses). Throughout all CMA-ES searches, spatially resolved local uncertainty, along with the loss, served as an indicator for structure stability. Importantly, after adding the structures from the exploration-based active learning in the (2 × 3) cell, Fig. 2(d), to the training data, the MLFF learned to avoid regions leading to the previously observed overlayer instability. Fig. S4 of the ESI† shows uncertainty trajectories for (2 × 3), (2 × 4), and (2 × 5) runs, where no local uncertainty exceeds 3*σ*_s_. Of particular interest is the (2 × 3) trajectory, which still illustrates that the Sr environment is the most uncertain, but does not escalate anymore (compare to Fig. 3). Importantly, this demonstrates the transferability of the model when extrapolating to reconstruction with larger unit cells.

**Fig. 4:**
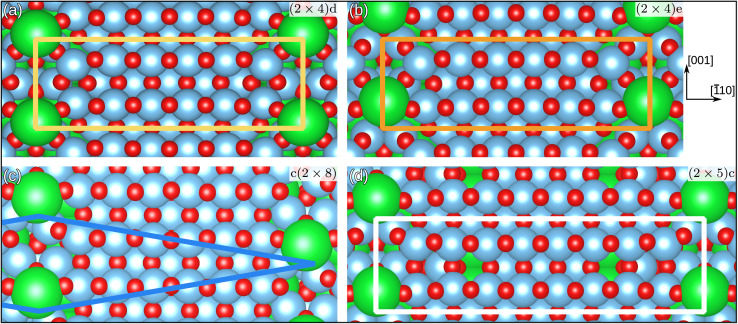
Top view of SrTiO_3_(110) overlayer candidates. (a) and (b) with (2 × 4) bulk periodicity. (c) With centered (2 × 8) periodicity and (d) with (2 × 5) periodicity. The orange and white rectangles and the blue rhombus indicate (parts of) the respective unit cells. Only half of the c(2 × 8) unit cell is outlined. See Fig. S11 of the ESI† for the full picture.

With this approach, we were able to discover the new candidate structures shown in Fig. 4, namely (2 × 4)d (yellow), (2 × 4)e (orange), c(2 × 8) (blue), and (2 × 5)c (white). All of these structures were then relaxed with VASP and will be compared to experiment in the following. They are available on Zenodo.^50^

### 4.2 Comparison to experiment

**Fig. 5:**
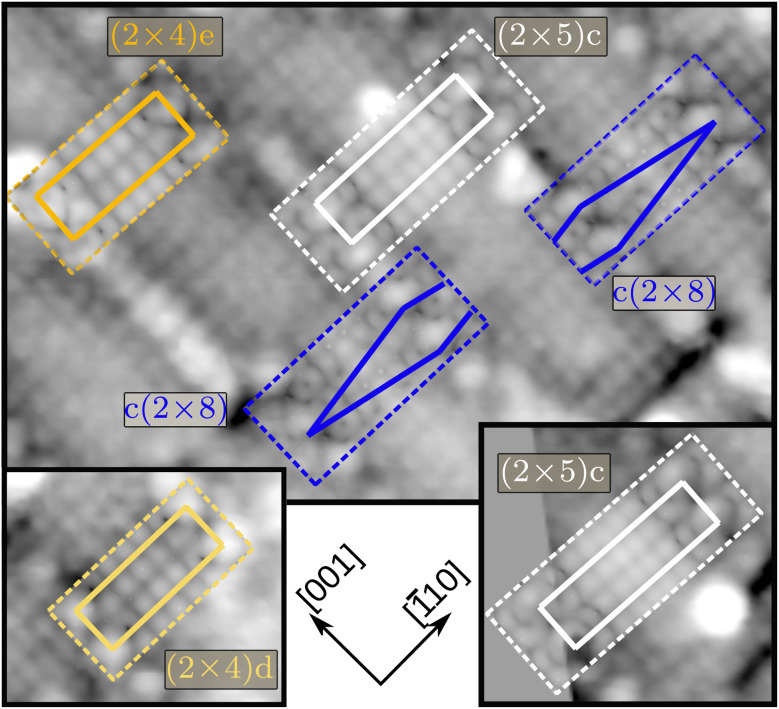
Different regions of the same STM measurement of Ti-rich SrTiO_3_(110), showing (2 × 4) (orange and yellow), c(2 × 8) (blue), and (2 × 5) (white) motifs. The colored dashed lines mark the border of simulated images, overlaid with 50% transparency, colored solid lines represent the model unit cells or parts thereof in the case of c(2 × 8). Imaging parameters: *V*_sample bias_ = +1.8 V, *I* = 0.04 nA. Simulated images were created using the Tersoff–Hamann approximation.^56^ Alternative versions of this figure, including without overlays, with fully opaque simulation images or the topmost atoms overlaid, as well as a larger cutout from the experimental image, are shown in the ESI as Fig. S1.†

Fig. 5 depicts a high-resolution STM image that illustrates how the preparation of Ti-rich surfaces results in a mixture of various surface structures. In this image, local symmetries with (2 × 4) (orange and yellow), (2 × 5) (white), and c(2 × 8) (blue) motifs can be observed. In the STM images of the Ti-rich SrTiO_3_(110) surfaces, the Sr atoms and TiO_*x*_ rows (henceforth referred to as “TiO ridges”) are visible as bright spots. In the cells shown in Fig. 5, the topmost TiO ridges tend to be aligned with the Sr atoms. This feature is incompatible with the STM image obtained from the published (2 × 5)b structure,^25^ where the TiO ridges are offset with respect to the Sr atoms.

In experiments, it is rare to encounter a surface composition that precisely matches the stoichiometry of a given thermodynamically stable surface reconstruction. Rather, the average composition observed in experiments often lies between two specific reconstructions, implying the coexistence of these reconstructions on the surface. Additionally, kinetic limitations may lead to the coexistence of even more surface reconstructions, with local variations in composition, while preserving the overall average surface composition.

To facilitate the investigation of these coexisting reconstructions, founder structures for SrTiO_3_(110)-(2 × 4) and -(2 × 5) cells were created as described in Section S3 of the ESI.† In short, they were generated by varying initial atom positions and adjusting the stoichiometry (addition or removal of TiO_2_ units). Details regarding the evolutionary searches, including the number of runs, population sizes *λ*, and step sizes *σ* are summarized in the Technical details. All newly proposed structures were tested by comparing the corresponding simulated STM images with the experimental data in Fig. 5. Important criteria for matching were the position of the Sr adatoms and their relative alignment to the TiO ridges.

For (2 × 5) systems, the initial placement of “TiO_2_ vacancies” resulted in distinct founders, with the vacancies positioned either in-line or out-of-line relative to the overlayer Sr in the [1̄10] direction. All sensible configurations resulting from these founders yielded significantly higher energies than the (2 × 5)b from ref. 25. However, an alternative candidate structure, (2 × 5)c (see Fig. 4), could be identified due to its distinct features. There, in contrast to (2 × 5)b, the overlayer Sr atom is aligned with a TiO ridge rather than being offset. While the energy difference between the two is Δ*E* = 208 meV per (1 × 1) bulk unit cell in favor of (2 × 5)b, the new (2 × 5)c clearly fits regions of the inhomogeneous surface, as shown in Fig. 5.

The investigation of SrTiO_3_(110)-(2 × 4) identified two stable surface structures, labeled (2 × 4)d and (2 × 4)e, both shown in Fig. 4. Although the difference in DFT energies between them is vanishingly small – only 2 meV per (1 × 1) bulk unit cell in favor of (2 × 4)d – the arrangement of the overlayer atoms is distinct. The most noticeable differences include the relative position of the overlayer Sr atom with respect to the TiO ridges and the resulting positional changes. Additionally, the centered unit cell c(2 × 8) was found as a candidate structure for explaining regions on the STM measurement showing a shift between Sr positions.

The newly proposed structures (2 × 4)d, (2 × 4)e, c(2 × 8), and (2 × 5)c thus provide previously missing atomistic models, which we successfully matched to the various experimentally observed motifs.

## 5 Summary and conclusions

We successfully integrated neural-network force fields with the covariance matrix adaptation evolution strategy to develop an accurate and transferable machine-learned force field suitable for the exploration of Ti-rich SrTiO_3_(110) surface reconstructions. The required training data were generated through an active learning workflow, which involved repeatedly performing CMA-ES runs on SrTiO_3_(110)-(2 × 2) and -(2 × 3) founder structures to gather uncertain and diverse data. During this process, invariant, descriptor-based NeuralIL committees were utilized for energy evaluation and uncertainty estimation. The collected data was then used to train an equivariant MACE model with learned representation suitable for production runs.

To fine-tune the training data in a further AL step, and thereby enhance model performance, we employed spatially resolved uncertainty estimation to identify underrepresented local environments which global uncertainty measures had failed to resolve. The resulting MLFF, MACE_full_, was trained on 2618 structures spanning SrTiO_3_(110)-(*n* × *m*), *n* ∈ {4, 5}, *m* ∈ {2, 3}.

We successfully identified two not previously reported candidates for stable (2 × 3) reconstructions. These structures were then used to extrapolate to (2 × 4) and (2 × 5) founder structures for evolutionary exploration. With this approach, we found new stable candidate structures for SrTiO_3_(110)-(2 × 4) and -(2 × 5), explaining different experimentally observed regions of the inhomogeneous Ti-rich surface. This method could be extended to other multi-element oxides featuring complex, composition-related, and possibly coexisting surface reconstructions characterized by large motifs.

## Data availability

The trained models, training and test data, and POSCAR files containing founders and results are available on Zenodo. This dataset also includes an example evolution script to be used in combination with CLINAMEN2 and MACE.^50^

## Conflicts of interest

There are no conflicts to declare.

## Supplementary Material

DD-003-D4DD00231H-s001

DD-003-D4DD00231H-s002

DD-003-D4DD00231H-s003
