# Supplementary figures and images for: Exploring inhomogeneous surfaces: Ti-rich SrTiO3(110) reconstructions via active learning
Source: Digit Discov. 2024 Sep 16;3(10):2137–45. doi: 10.1039/d4dd00231h (PMC11443185; doi:10.1039/d4dd00231h)

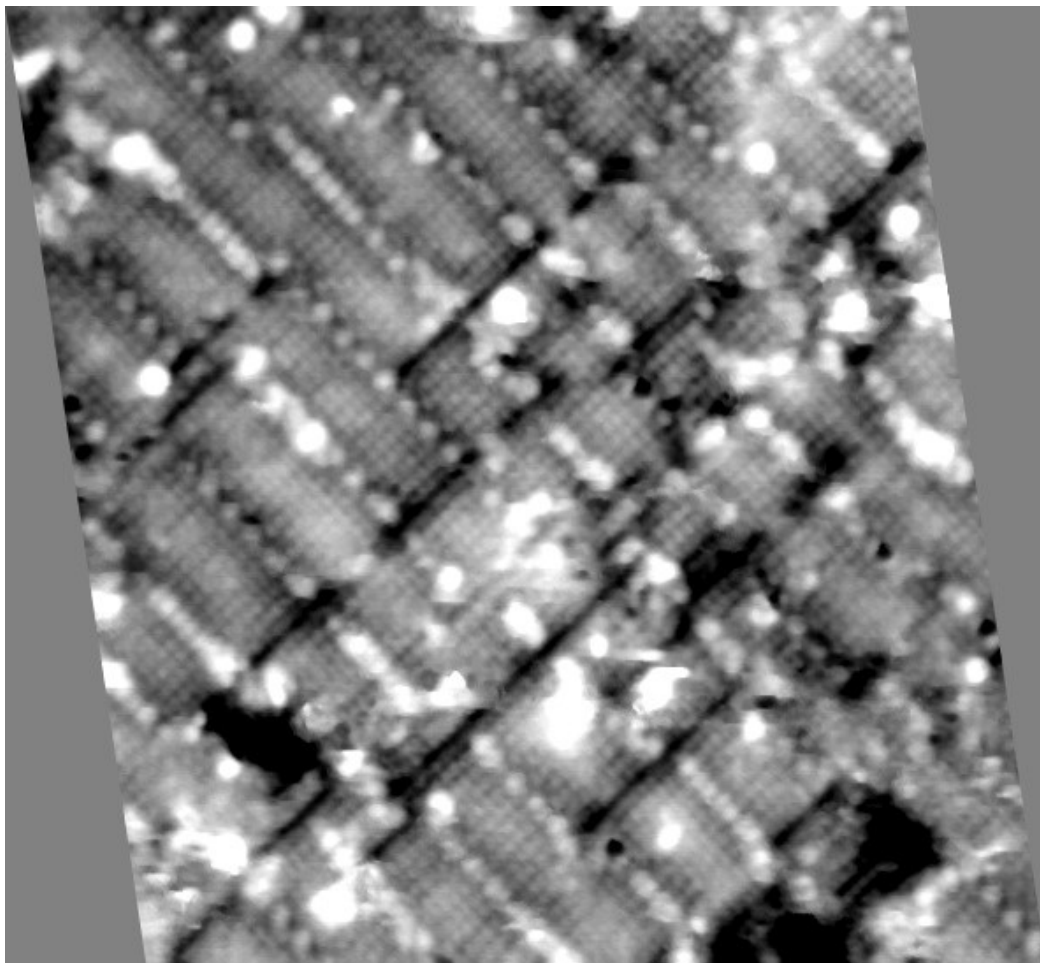

Supplement: DD-003-D4DD00231H-s001 [file DD-003-D4DD00231H-s001.pdf]
