# Supplementary material for: Exploring inhomogeneous surfaces: Ti-rich SrTiO3(110) reconstructions via active learning
Source: Digit Discov. 2024 Sep 16;3(10):2137–45. doi: 10.1039/d4dd00231h (PMC11443185; doi:10.1039/d4dd00231h)
Supplement: DD-003-D4DD00231H-s002 [file DD-003-D4DD00231H-s002.pdf]

Supplementary Information (SI) for Digital Discovery.
This journal is © The Royal Society of Chemistry 2024

Supplementary Information for Exploring Inhomogeneous Surfaces: Ti-rich $SrTiO_3(110)$ Reconstructions via Active Learning

Ralf Wanzenböck,[a] Esther Heid,[a] Michele Riva,[b] Giada Franceschi,[b] Alexander M. Imre,[b] Jesús Carrete,[c] Ulrike Diebold,[b] and Georg K. H. Madsen*[a]

S1. STM comparison

At the bottom of Fig. S1, panel (e), the motifs explained by the newly identified $SrTiO_3(110)$ unit cells are marked on a part of the experimental STM image. The original STM image has been included as a separate file (2017-11-13.mul%12 - BG_Scr_NL_L_scale.png). Fig. 5 of the main manuscript is reproduced four times in Fig. S1: (a) no overlaid simulated STM, (b) fully opaque simulated STM overlaid, (c) top-most atoms of unit cell overlaid, and (d) top-most atoms of unit cell overlaid and simulated STM on top of the experimental image.

An animation showing the simulated STM overlay transitioning from transparent to opaque can be found in a separate file (stm_with_sim.avi).

[a] Institute of Materials Chemistry, TU Wien, 1060 Vienna, Austria. E-mail: georg.madsen@tuwien.ac.at
[b] Institute of Applied Physics, TU Wien, 1040 Vienna, Austria.
[c] Instituto de Nanociencia y Materiales de Aragón, CSIC-Universidad de Zaragoza, 50009 Zaragoza, Spain.

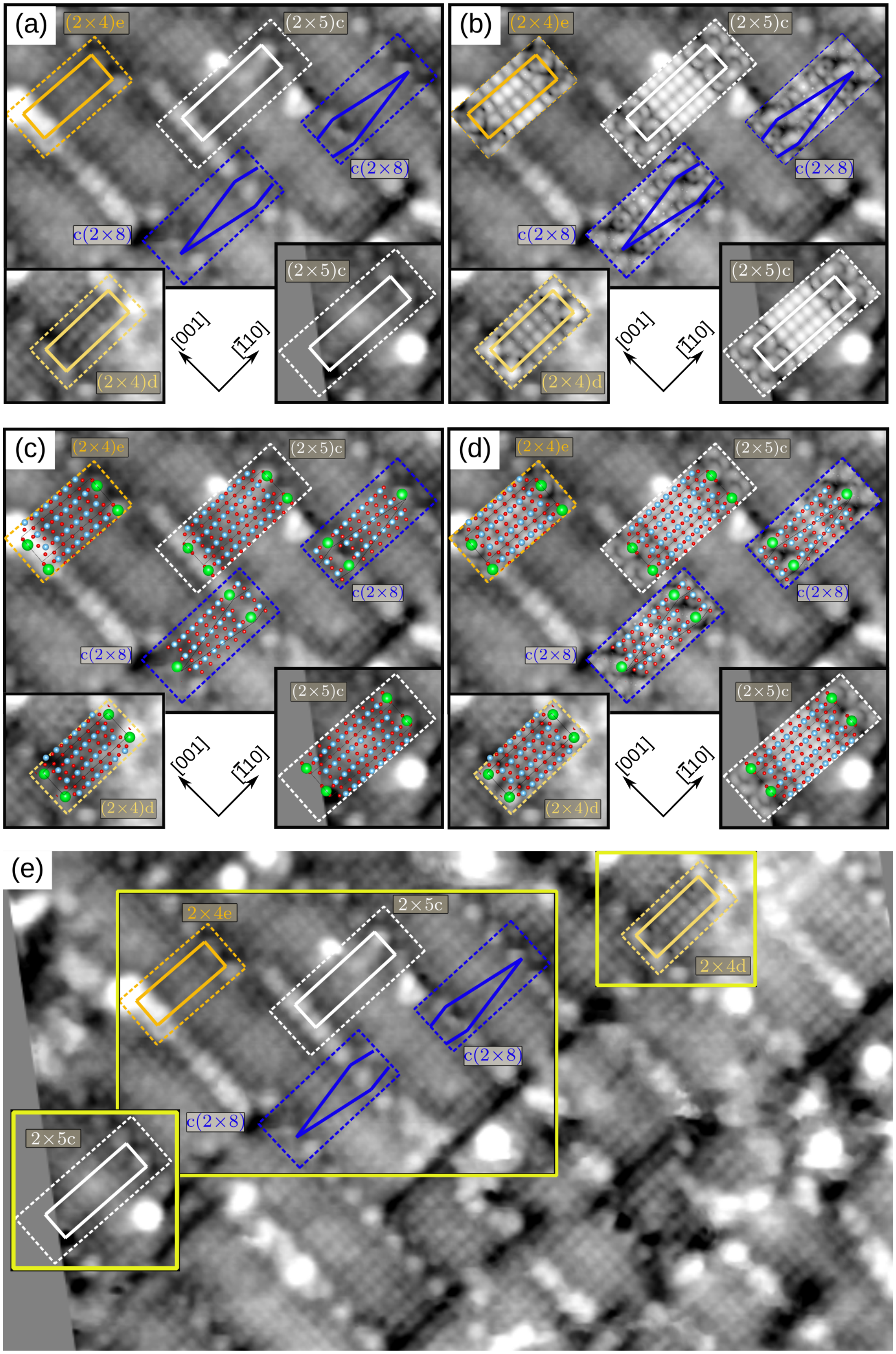

Fig. S1 Part of an experimental STM image of Ti-rich $SrTiO_3$(110) showing (2×4) and (2×5) motifs. Panels (a)-(d) reproduce Fig. 5 of the main manuscript with different details overlaid and solid lines outlining unit cells. In (e) the position of the motifs is highlighted instead of combining cutouts.

S2. Uncertainty estimation

Figure S2 shows the correlation between force uncertainty estimate and true force error in good agreement.

As discussed in the main text of the manuscript, we built on data from Ref. 1 and used a ten-member NeuralIL committee in an active learning approach to collect the required data[2,3]. Data was collected by evaluating the aggregated force uncertainty for each structure along evolution trajectories and selecting accordingly. In Fig. S3, we show the strong agreement of these uncertainty estimates for the high-uncertainty regions of 20 (2×3) $SrTiO_3(110)$ CMA-ES[4] runs, between a ten-member NeuralIL committee and a five-member MACE[5] committee. Both committees were trained on the same data.

Figure S4 illustrates the behavior of spatially-resolved local uncertainties along evolution trajectories[6], highlighting the stability of the $MACE_{full}$ model for different system sizes. The original five-member MACE committee was used to calculate these uncertainties. With that we can show that evolutions performed using $MACE_{full}$ still visit regions where the previous model is locally uncertain, but that the unphysical configurations no longer come up.

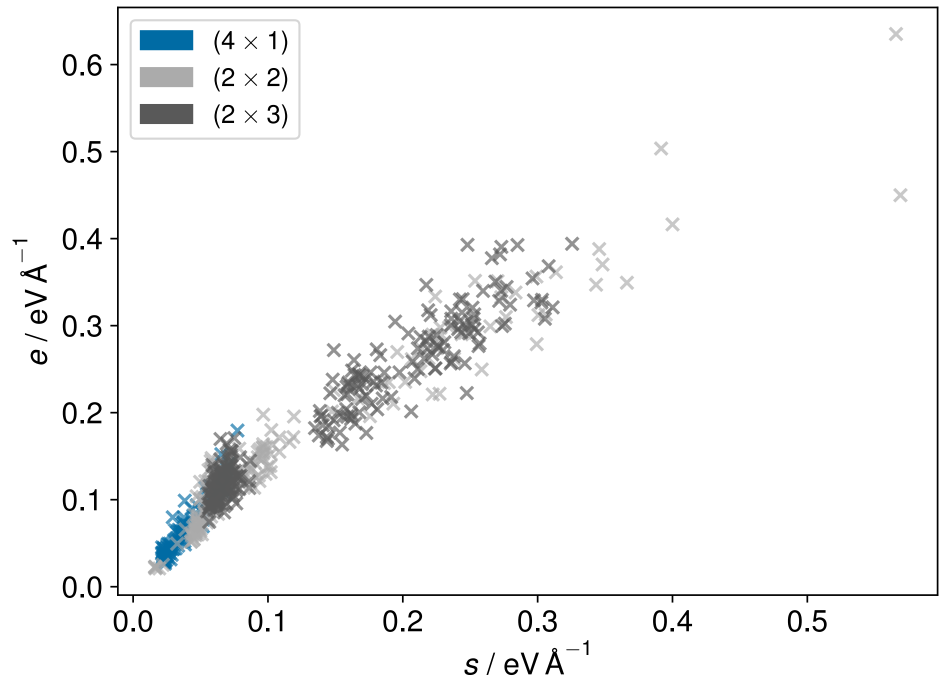

Fig. S2 Parity plot showing the true error e vs the MACE uncertainty estimation s on the test set explained in the main manuscript, containing $SrTiO_3$(110)-($n\times1$) -(2×2), and -(2×3) structures.

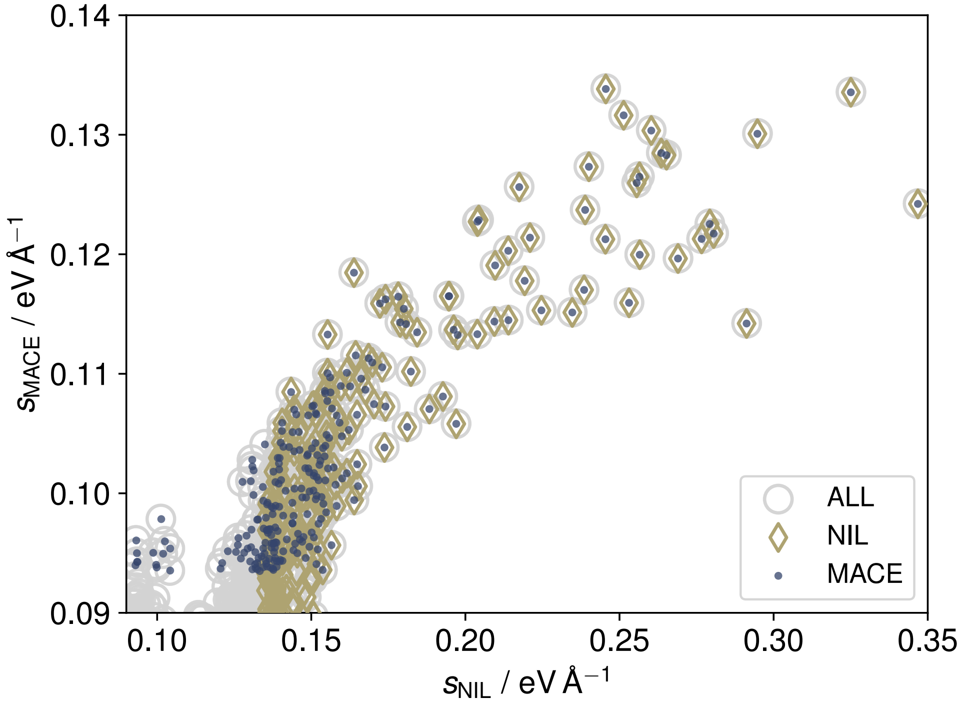

Fig. S3 Comparison of high-uncertainty estimates between NeuralIL and MACE. The gray circles show the mean configurations from 20 (2×3) $SrTiO_3$(110) CMA-ES runs, zooming in on the high-uncertainty region and illustrating the good agreement between the aggregated force uncertainty estimates from a MACE committee (blue dots) and from a NeuralIL committee (golden rhombuses).

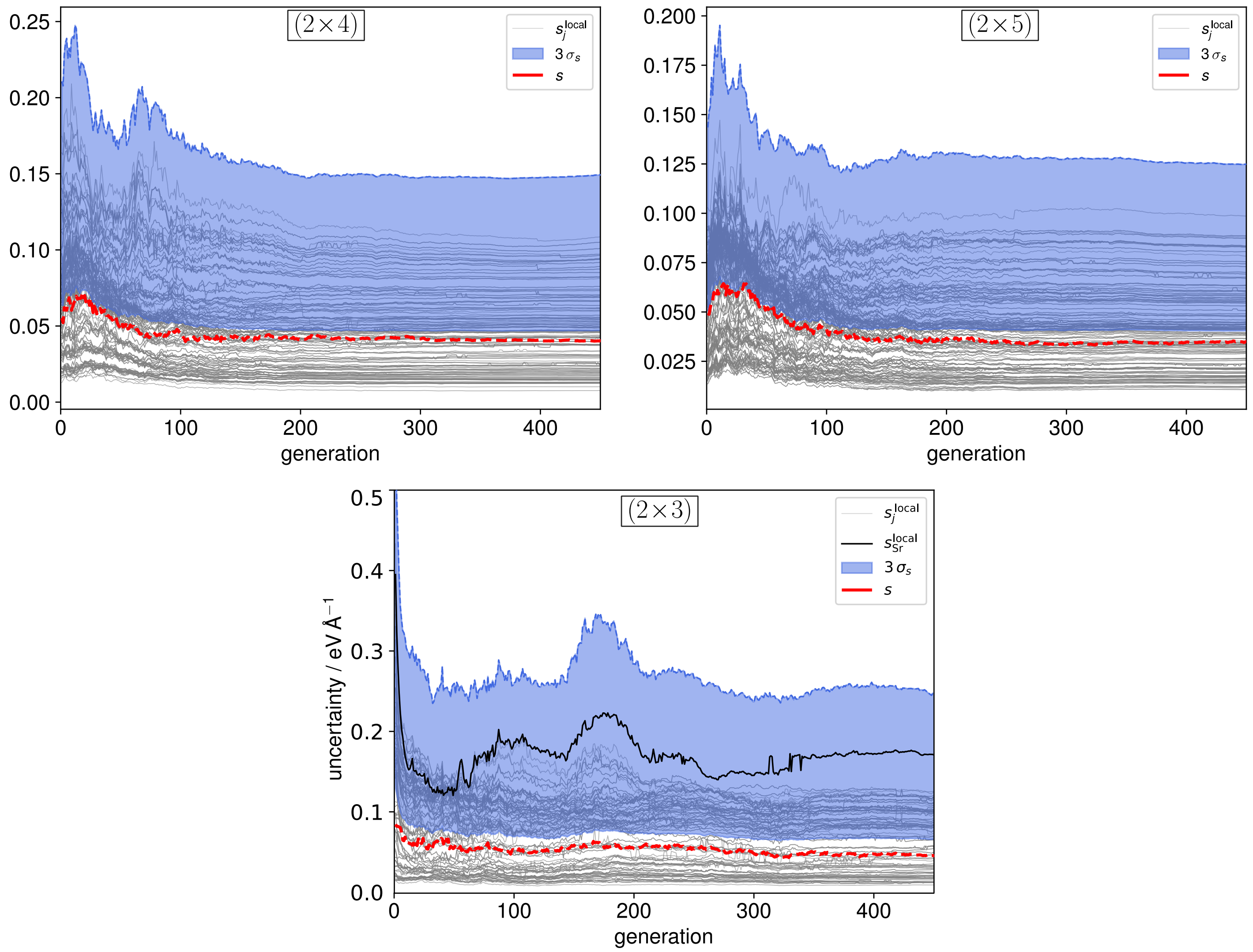

Fig. S4 Spatially-resolved local uncertainties for the mean of the first 450 generations of three CMA-ES trajectories, $SrTiO_3$(110)-(2×3), (2×4) and (2×5), respectively. Gray lines indicate the locally aggregated uncertainty s_j^{local} of each atom. The overall mean of these local uncertainties, i.e. the global structure uncertainty s, is depicted as a dashed red line, with the region representing three times the standard deviation σ_s shaded in blue. In the bottom panel, the solid black line highlights the local uncertainty associated with the overlayer Sr atom for comparison with the main manuscript.

S3. Creating founder structures

Figure S5 illustrates how attachment- and overlayer of the $SrTiO_3(110)$-(2×3)c cell were extrapolated to fit onto the larger bulk-cut slabs. The (2×3)c structures were treated in units of (2×1) bulk unit cells, with the first block reproduced identically on top of the bulk-cut slabs at $m = 1$. Next, the atoms positioned at the center of the original unit cell in the $[\bar{1}10]$ direction were reproduced at the corresponding center of each larger cell. While this step was trivial for (2×5), differences in bulk-like and, therefore, attachment layers between odd and even m needed to be considered for the (2×4). Subsets of the transplanted attachment- and overlayer atoms were then repeated to fill the remaining space between the first block and the center. Finally, mirror symmetry along $[\bar{1}10]$ was applied to complete the overlayers. The founder structures depicted in Fig. S6 resulted from this extrapolation approach. There, different stoichiometries of the (2×4) and variation in TiO_2 vacancy positions in (2×5) are shown. Fig. S7 presents the generic (2×3) founder featuring an overlayer of copied and shifted bulk atoms with enforced mirror symmetry in $[\bar{1}10]$ direction. The slab setup is further illustrated in Fig. S8.

Table S1 lists the structures that are referenced via their label in the main manuscript. All of these structures are polarity compensating as a result of an appropriate excess of oxygen atoms, corresponding to a net $-2e$ charge in the surface layer.

Table S1 Periodicity, stoichiometry, and building-block stoichiometry of the overlayers of the $SrTiO_3(110)$-$(m \times n)$ reconstructions used as training data or presented as results in the main manuscript. The right-most column gives the atom count of the full slab. Note that while these stoichiometries can be identical, the atomic positions need to be taken into account for the full picture.

**Periodicity**	**Stoichiometry**	**Building blocks**	**#atoms (full)**
(2×3)a [7]	$Sr_2Ti_{17}O_{42}$	$(SrO)_2(TiO_2)_{17}(O)_6$	260
(2×3)b [7]	$SrTi_{18}O_{43}$	$(SrO)(TiO_2)_{18}(O)_6$	262
(2×3)c	$SrTi_{18}O_{43}$	$(SrO)(TiO_2)_{18}(O)_6$	262
c(2 × 6)	$SrTi_{18}O_{43}$	$(SrO)(TiO_2)_{18}(O)_6$	262
(2×4)b [7]	$SrTi_{25}O_{59}$	$(SrO)(TiO_2)_{25}(O)_8$	354
(2×4)c	$Sr_1Ti_{26}O_{61}$	$(SrO)(TiO_2)_{26}(O)_8$	360
c(2 × 8)	$Sr_1Ti_{25}O_{59}$	$(SrO)(TiO_2)_{25}(O)_8$	354
(2×5)b [7]	$SrTi_{32}O_{75}$	$(SrO)(TiO_2)_{32}(O)_{10}$	446
(2×5)c	$SrTi_{32}O_{75}$	$(SrO)(TiO_2)_{32}(O)_{10}$	446
(2 × 2) [7]	$Ti_{14}O_{32}$	$(TiO_2)_{14}(O)_4$	184
(4 × 1) [8]	Ti_6O_{16}	$(TiO_2)_6(O)_4$	136
(5 × 1) [8]	Ti_7O_{19}	$(TiO_2)_7(O)_5$	167

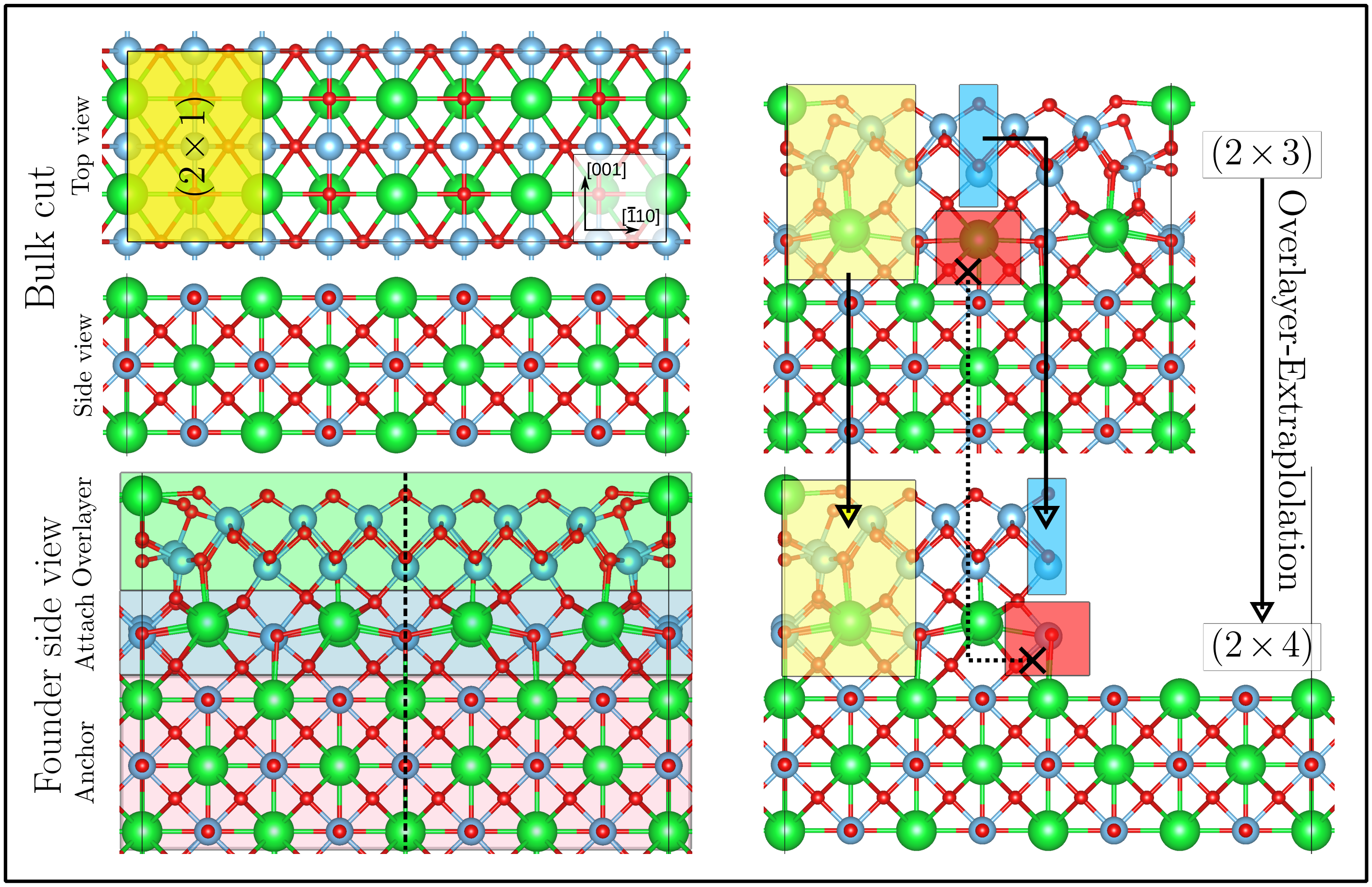

Fig. S5 The top-left subplot shows a top and side view of a bulk-cut $SrTiO_3$(110)-(2×4) slab, reduced to minimal anchor layers. The dark-yellow box indicates a (2×1) block. On the bottom left, a side view of the extrapolated (2×4)c founder is shown. Background colors highlight the separation into over-, attachment- and anchor-layers. The dashed black line indicates a mirror plane. The right-hand-side of the figure illustrates the extrapolation of over- and attachment-layer from (2×3) to (2×4). The light-yellow highlight indicates the attachment- and overlayer on top of a (2×1) bulk block, which is transplanted $1:1$. While the blue rectangle indicates which mirror-plane atoms can be reproduced on the larger surface, a problematic region is shown in red. Due to the difference in multiplicity along $[\bar{1}10]$, the attachment needs to be carefully adjusted to keep a sensible configuration. Note that in a refinement step, atoms near the projected mirror plane are put directly on it by design.

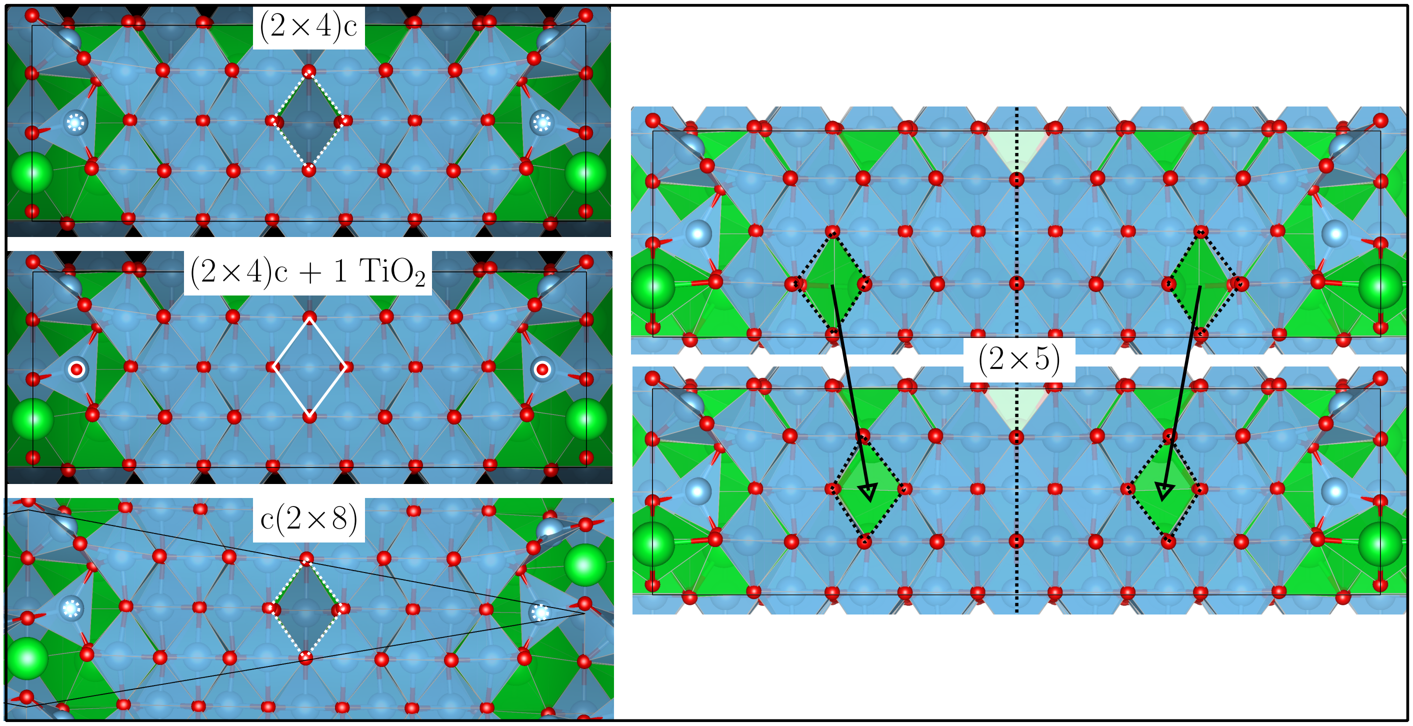

Fig. S6 The left column presents three founders for $SrTiO_3$(110)-(2×4) systems, including the centered unit cell c(2×8). White dotted circles and lines on (2×4)c and c(2×8) indicate the "vacancies" when compared to the additional Ti and 2 O atoms present in the center pane. On the right-hand-side, two $SrTiO_3$(110)-(2×5) founders with identical stoichiometry, but different "TiO_2-vacancies" are shown. The vertical dotted line highlights a mirror plane.

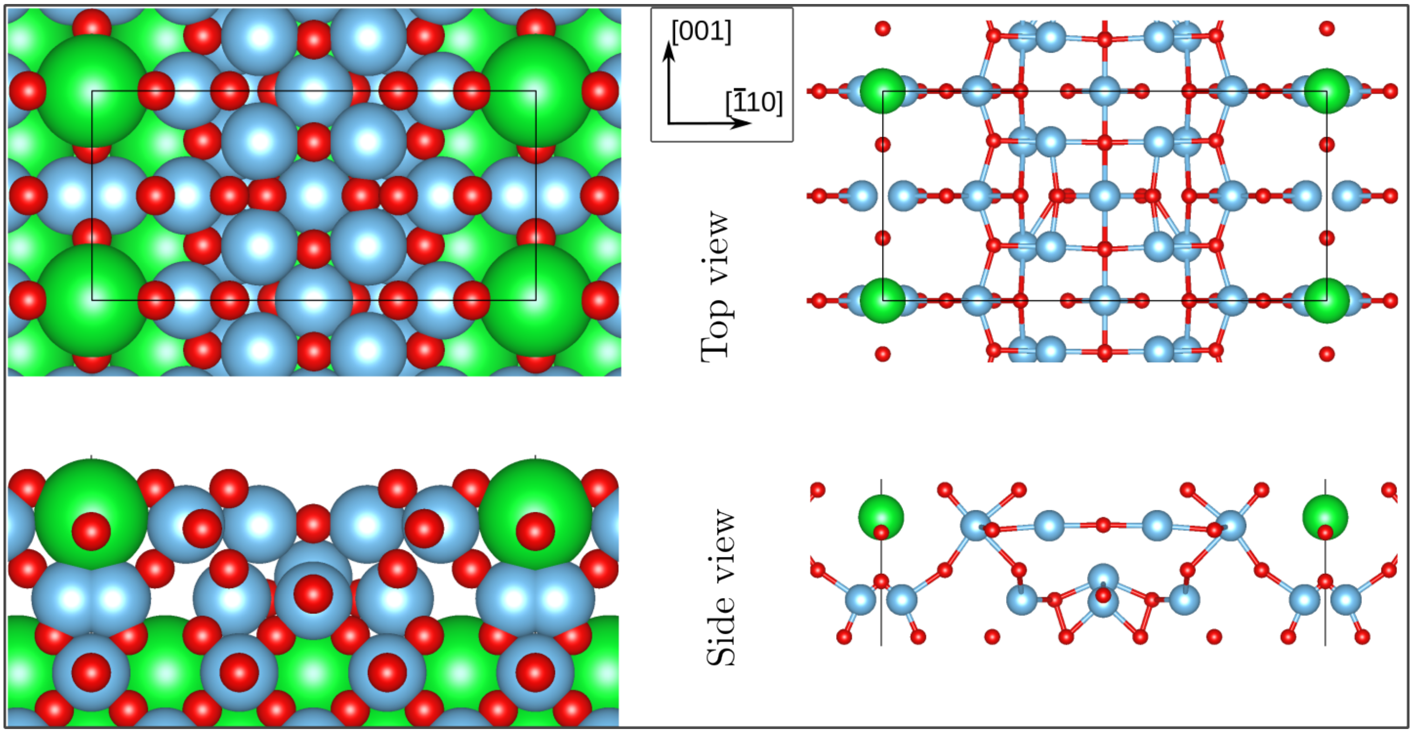

Fig. S7 Top and side view of the founder "POSCAR_founder_generic_2x3". The left column shows a space-filling representation, the right-hand-side depicts a ball-and-stick model, which is reduced to only include the overlayer atoms. Unit cells are shown as solid black lines.

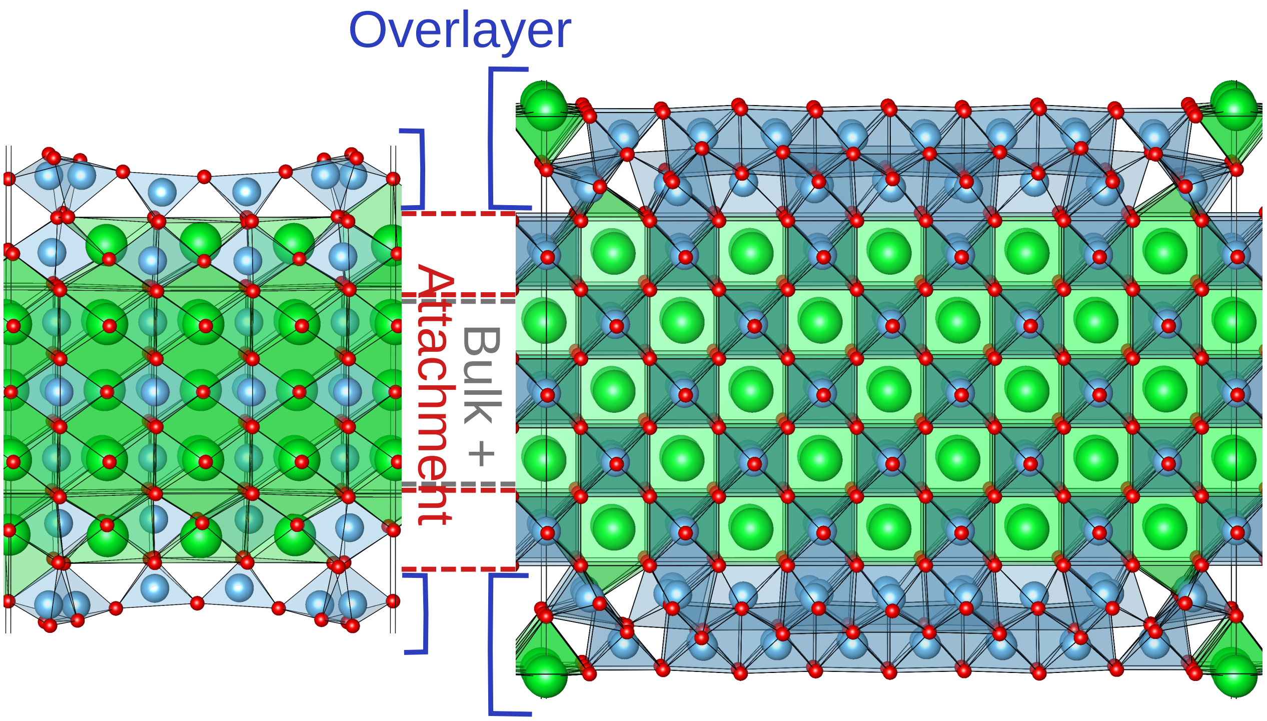

Fig. S8 An illustration of the slab setup on two examples, (4×1) (left) and (2×4)b (right). The bulk-like, fixed "anchor" layers are between the gray dashed lines. The surface structure consist of the attachment-layer (red lines) and the overlayer (blue brackets). Both sides of the slab along the surface normal are symmetrical. In this study, the CMA-ES manipulates the positions of the atoms within the attachment-layer and overlayer on one side.

S4. Model performance

Table S2 compares the performance of the fully trained NeuralIL and MACE models on three test sets, showing a significant improvement.

Table S2 : f_{MAE} in $\mathrm{meV\AA^{-1}}$ for both NeuralIL and MACE on the three test sets.

**Model**	**($n\times1$)**	**(2×2)**	**(2×3)**
NeuralIL	147.21	407.49	480.90
MACE	62.00	205.00	220.00

Fig. S9 shows the performance of the final MACE model on a mixed test set of $SrTiO_3(110)$-(4×1), (2×2), and (2×3) structures. The energy is expressed in eV per (1×1) bulk unit cell and is calculated via

$$E_{\mathrm{buc}} = \frac{E_{\mathrm{slab}}}{2\,n_{\mathrm{buc}}}, \tag{1}$$

from the energy of the slab E_{slab} and n_{buc} the number of (1×1) bulk unit cells covered by the overlayer.

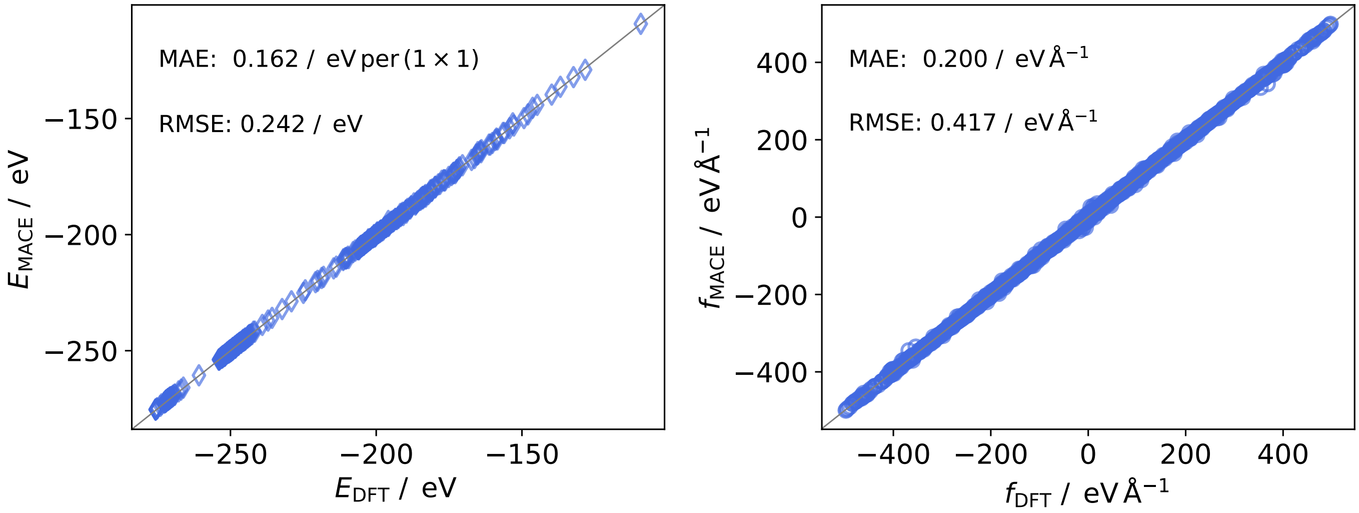

Fig. S9 Parity plots for the energies and force components showing the performance of $MACE_{full}$ on a mixed test set of 524 $SrTiO_3(110)$ structures:98 (4×1), 155 (2×2), and 271 (2×3). The test configurations cover a very diverse range of structures, including unphysical structures encountered in early generations.

S5. Additional views of selected structures

Figures S10 and S11 depict selected structures from the main manuscript as viewed from different angles.

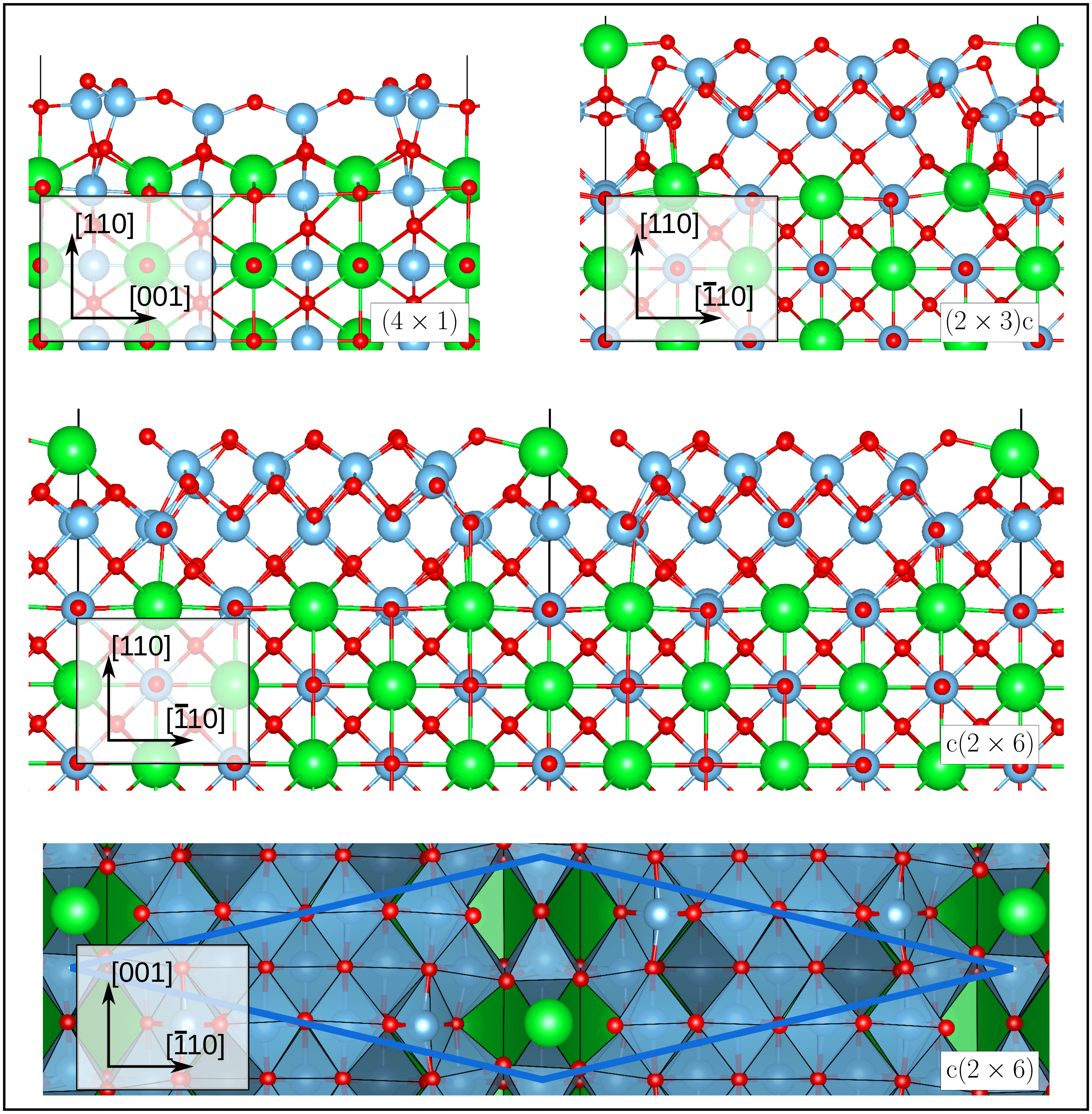

Fig. S10 Side views for (4×1) and (2×3)c in the top row. The middle and lower plots both show c(2×6), in a side view and from the top. The border of the full unit cell of c(2×6) is outlined in blue.

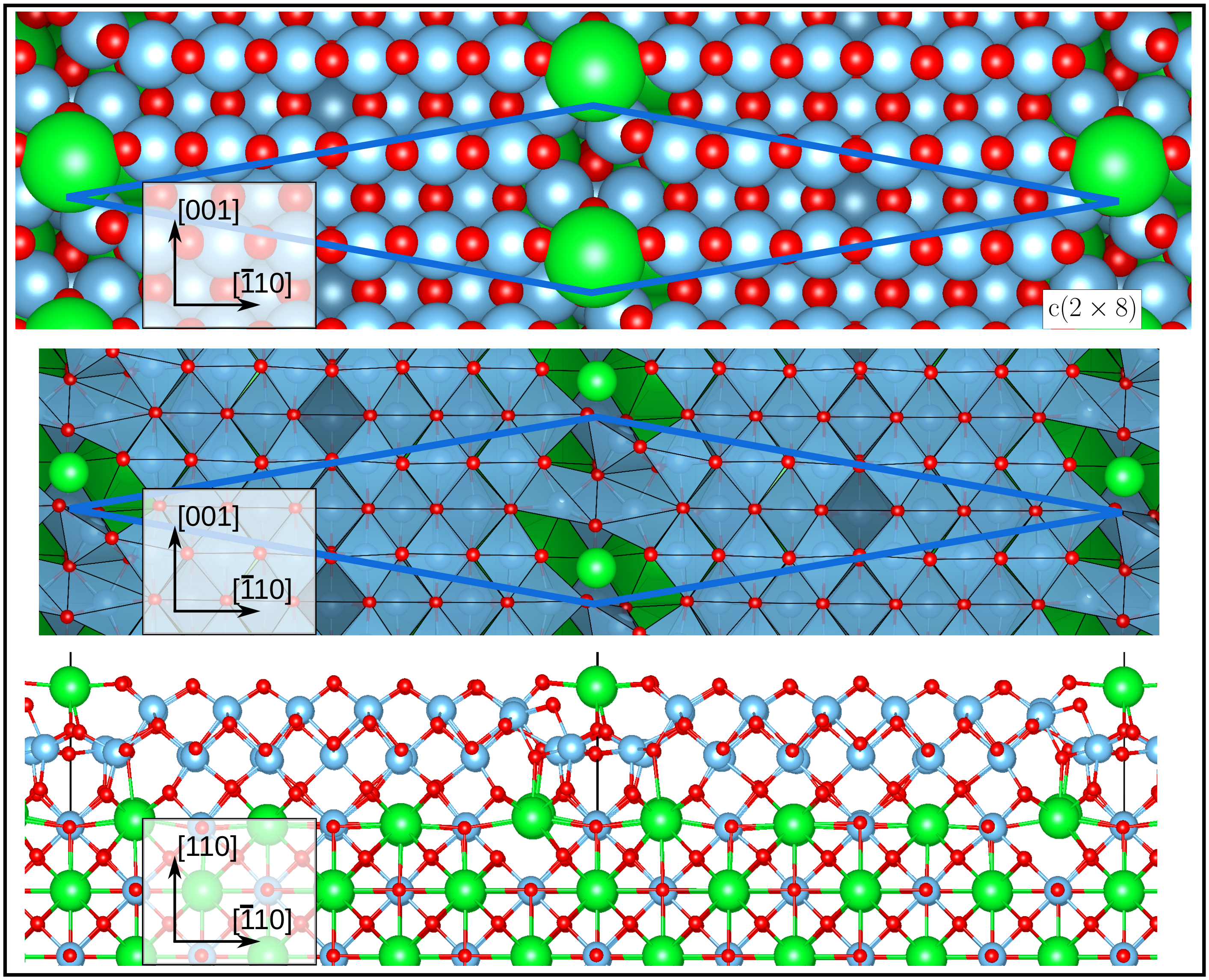

Fig. S11 Top views (space filling and polyhedra) of c(2×8). The border of the full unit cell is outlined in blue. The bottom row shows a side view of the same structure.

References

1 R. Wanzenböck, M. Arrigoni, S. Bichelmaier, F. Buchner, J. Carrete and G. K. H. Madsen, *Digit. Discov.*, 2022, **1**, 703–710.

2 H. Montes-Campos, J. Carrete, S. Bichelmaier, L. M. Varela and G. K. H. Madsen, *J. Chem. Inf. Model.*, 2021, **62**, 88–101.

3 J. Carrete, H. Montes-Campos, R. Wanzenböck, E. Heid and G. K. H. Madsen, *J. Chem. Phys.*, 2023, **158**, 204801.

4 N. Hansen, *The CMA Evolution Strategy: A Tutorial*, 2016, arXiv:1604.00772 [cs.LG], 2016.

5 I. Batatia, D. P. Kovacs, G. Simm, C. Ortner and G. Csányi, *Adv. Neural. Inf. Process. Syst.*, 2022, **35**, 11423–11436.

6 E. Heid, J. Schörghuber, R. Wanzenböck and G. K. H. Madsen, *J. Chem. Inf. Model., JCIM*, 2024, **64**, 6377–6387.

7 Z. Wang, A. Loon, A. Subramanian, S. Gerhold, E. McDermott, J. A. Enterkin, M. Hieckel, B. C. Russell, R. J. Green, A. Moewes, J. Guo, P. Blaha, M. R. Castell, U. Diebold and L. D. Marks, *Nano Lett.*, 2016, **16**, 2407–2412.

8 J. A. Enterkin, A. K. Subramanian, B. C. Russell, M. R. Castell, K. R. Poeppelmeier and L. D. Marks, *Nat. Mater.*, 2010, **9**, 245–248.
